# Fat suppression for coronary MR angiography at 3T: 2 point Dixon versus Spectral Presaturation with Inversion Recovery (SPIR)

**DOI:** 10.1186/1532-429X-15-S1-E9

**Published:** 2013-01-30

**Authors:** Markus Henningsson, Peter Boernert, Peter Koken, Rene M Botnar

**Affiliations:** 1 Division of Imaging Sciences and Biomedical Engineering, King's College London, London, UK; 2 Philips Research, Hamburg, Germany

## Background

The coronary arteries are embedded in epicardial fat. To improve visualization in coronary MR angiography (CMRA) fat suppression techniques such as Spectral Presaturation with Inversion Recovery (SPIR) are often employed. Recently, water-fat separation using the multi-echo 2 point Dixon method has gained increasing interest as it allows decomposing the MR signal into a water and fat image during image reconstruction [Eggers, MRM 2011]. While the water image provides a coronary angiogram the fat image may also contain clinically useful information and could be used for epicardial/pericardial fat quantification [Koken, ISMRM, 2011]. The purpose of this study was to compare SPIR and 2 point Dixon for coronary vessel delineation for the first time on a 3T clinical scanner.

## Methods

Imaging parameters for the T2 prepared, navigator gated CMRA Dixon sequence were; field-of-view = 300 x 300 x 100 mm^3^, resolution = 1.2 x 1.2 x 1.2 mm^3^, TR/TE1/TE2 = 4.0/1.36/2.4 ms, α=20°, and SENSE = 2 in phase encoding direction with a nominal scan time of 7:03 mins assuming a heart-rate of 60 bpm. The images were acquired in the coronal plane with read-out in feet-head direction. A 32 channel cardiac coil was used for signal detection. The imaging parameters for the acquisition using SPIR were identical, however only one echo (TE1) was acquired. The order of the 2 point Dixon and SPIR scan was randomized and five healthy volunteers were examined on a 3T Philips scanner. For comparison of coronary artery delineation the images were reformatted and the vessel sharpness of the right coronary artery (RCA) and the left anterior descending artery (LAD) were quantified.

## Results

**Figure 1:**
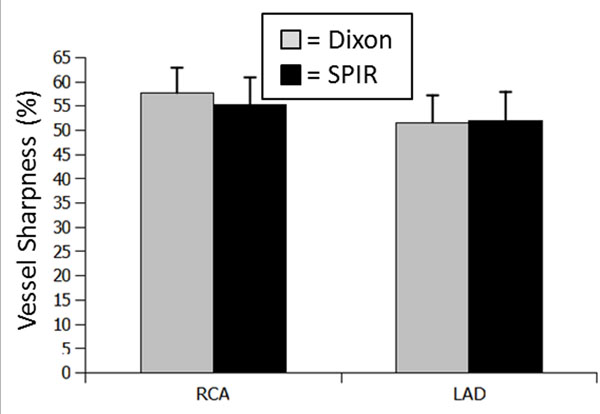
Vessel sharpness of right coronary artery (RCA) and left anterior descending (LAD) for the Dixon and spectrally selective inversion recovery (SPIR) fat suppression methods.

**Figure 2:**
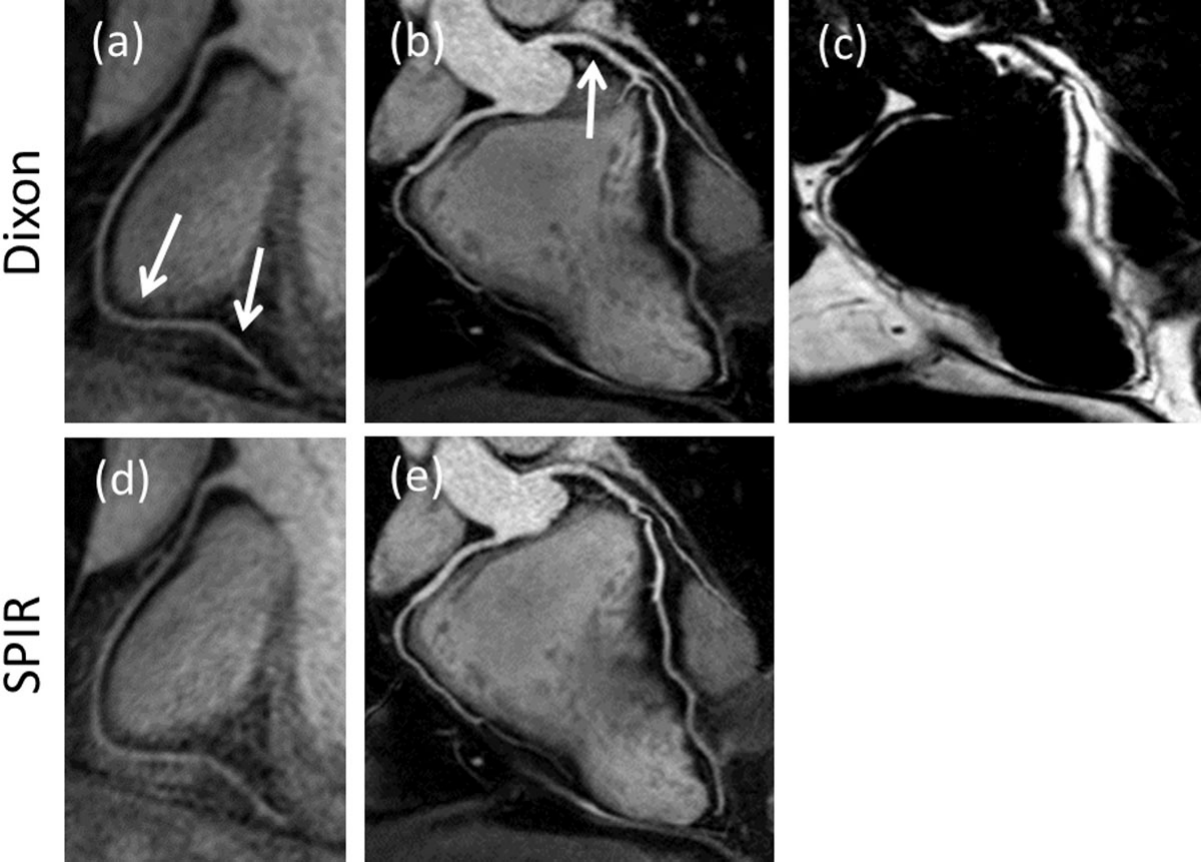
CMRA images of the RCA from one healthy volunteer using Dixon (a) and SPIR (d). Arrows highlight areas of improved fat suppression. RCA and LAD images from another healthy volunteer acquired with Dixon (b) and SPIR (e), where the additional fat image (c) from the Dixon acquisition is also shown.

Figure 1 shows the vessel sharpness measurements for the RCA and LAD for the two fat suppression methods. No statistically significant differences were found (P>0.05), however a noticeably improved fat suppression could be observed in a number of subjects in the distal RCA. One such example is shown in Figure 2 (a - Dixon, d - SPIR). In general, both fat suppression techniques produced excellent CRMA images and an example is shown in Figure 2 (b - Dixon, e SPIR). However, the Dixon method allowed generating a fat image in addition (Figure 2 (c)).

## Conclusions

We have shown that CMRA can be acquired with excellent fat suppression and image quality at 3T. Although no statistical significant difference were found between SPIR or Dixon in terms of coronary vessel sharpness in this small study, however Dixon provided a more robust fat suppression as it is less susceptible to B0 and B1 inhomogeneities which makes it favourable at higher field strengths (>1.5T). Furthermore, the additional fat image may provide novel clinical information.

## Funding

British Heart Foundation: RG/12/1/29262.

